# Sleeping Patterns of Afghan Unaccompanied Asylum-Seeking Adolescents: A Large Observational Study

**DOI:** 10.1371/journal.pone.0056156

**Published:** 2013-02-14

**Authors:** Israel Bronstein, Paul Montgomery

**Affiliations:** 1 Centre for Evidence Based Intervention, Department of Social Policy and Intervention, University of Oxford, Oxford, United Kingdom; 2 Louis and Gabi Weisfeld School of Social Work, Bar-Ilan University, Ramat Gan, Israel; University of California, San Francisco, United States of America

## Abstract

Unaccompanied asylum-seeking children (UASC) have experienced multiple traumas and are a high-risk group for posttraumatic stress disorder (PTSD). The effects of trauma are known to be associated with sleep problems; indeed sleeping problems are core features of PTSD. However, there has been no systematic research examining the sleep of this high risk group of children. This study presents the first evidence on the sleeping patterns of Afghan UASC living in the UK. A total of 222 male Afghan children, aged 13–18, were interviewed using validated self-report questionnaires measuring sleeping patterns and PTSD. Overall, UASC patterns for bed time and rise time appear acculturated to the country of asylum. Mean UASC sleep onset latency scores were approximately 20 minutes greater compared with normative scores, which may be a reflection of UASC pre-migration and post-migration experiences. As expected, UASC who screened above the clinical cut-off for PTSD reported significantly greater sleep onset latency, increased nightmares, and less total sleep time compared to the non-PTSD group. The results may be of particular interest to clinicians given that, compared to screening for PTSD, screening for sleep problems may be a less culturally disputed form of initial assessment indicating distress in UASC. Similarly, the field of UASC and refugee child interventions is largely focused on trauma, yet sleep may provide a novel avenue for equally or more effective treatment.

## Introduction

Unaccompanied asylum-seeking children (UASC) are young people under the age of 18 who are separated from their parents and have made a claim for political asylum under the 1951 Convention Relating to the Rights of Refugees (Geneva Convention) [1]. Between the years 2000–2009 the UK received approximately 30,000 UASC, the majority of whom came from Afghanistan [2]. UASC may experience a number of traumatic events, including exposure to extreme violence, terror, physical and/or sexual abuse, and rape [3]–[5]. Systematic reviews on the mental health of refugee children indicate prevalence estimates for posttraumatic stress disorder (PTSD) from 19–54% and for depression from 3–30%, with UASC reporting significantly greater symptoms compared to their accompanied peers [6]–[8]. There is an on-going debate however, about the appropriateness of applying Western mental health concepts such as PTSD to UASC populations [9]. One potential way of overcoming the conceptual arguments is through the assessment of sleep problems within this population. Subjective sleep problems, such as difficulty initiating sleep and nightmares, are common in the aftermath of traumatic events, e.g. [10], [11]. Sleep problems are also considered core features of PTSD and comprise part of the DSM-IV criteria for the disorder in the re-experiencing and hyper-arousal clusters [12]. For assessment purposes, the assessment of sleep may be less culturally laden compared to screening measures for PTSD, and could provide quick and helpful insight into the distress experienced by UASC.

Within this group of children, sleep problems should not only be considered as symptoms of possible PTSD but as problems themselves, given they can lead to a range of daily functional impairments in memory, concentration, attention, motor performance, academic performance, and behaviour [13]–[15]. In children specifically, sleep problems may influence cognitive and behavioural functions, and lead to increased fatigue, sleepiness, and slower reaction times [16]. A greater understanding of UASC sleep patterns and problems may provide a more consensual approach to the assessment and possible treatment of distress through the understanding of sleep as a symptom. Furthermore, there may be many UASC who are not experiencing PTSD but who are experiencing sleep problems, suggesting the risk for further problems developing over time.

Surprisingly, despite the importance of sleep functioning and the likely impact of previously experienced trauma, sleep remains one of the least researched aspects of UASC well-being. Sleep problems tend to receive only minor attention, primarily in terms of nightmares and difficulty sleeping, and only in a small number of qualitative studies about UASC [17]–[20]. In the wider literature on refugee children, Montgomery and Foldspang (2001) interviewed the parents of 311 accompanied refugee children, aged 3–15, who were from a range of countries and living in Denmark [21]. Parents were asked a number of questions about their child’s sleep, health, history of exile, and pre-migration experiences of war conditions. Approximately one-fifth of the children reportedly experienced frequent nightmares, problems falling asleep, and problems staying asleep. There are key contextual differences between UASC and these refugee children that greatly limit the generalisability of their study to the wider UASC population. Specifically, UASC are separated from their primary caregiver and have a liminal asylum status (i.e. uncertainty about their future to remain in the country of asylum).

This is the first study to investigate sleeping patterns of UASC. The objectives are (i) to understand the general patterns of UASC sleep and (ii) to investigate the relationship between these patterns and PTSD within this population.

## Materials and Methods

### Ethics Statement

This study was approved by the Central University of Oxford Research Ethics Committee (CUREC). All participants provided written informed consent and all data were analysed anonymously.

### Participants and Sampling

This is a cross-sectional, observational study. Afghan UASC were recruited from a single London local authority in 2010. Eligibility criteria indicated that young people had to be from Afghanistan, under the age of 18, unaccompanied or separated from their primary caregiver, claiming asylum alone, and in the care of local authority social services. Young people were excluded if they were living with any primary caregiver or if their age was disputed by the UK Border Agency or local authority social service.

A total of sample 326 UASC met the inclusion criteria. A three-stage process was used to obtain informed consent. All eligible UASC were posted documents in Dari, Pashto, and English, which included an opt-out letter, information about the research information, and consent forms. A period of 14 days was provided for UASC to return Opt-Out letters, following which initial contact was made through a trained multi-lingual recruitment officer. This officer read aloud information forms and answered questions before seeking the verbal consent of the young person. Data collection sessions were then arranged and conducted at a local college or meeting place of the young person’s choosing. On the day of data collection, the research assistant, with the aid of an interpreter, read the information sheets and consent forms to the UASC. Questions about the research and any matters of confidentiality were encouraged in a supportive environment prior to the youth providing written consent. Informed consent and full participation was provided by 222 UASC, 19 opted out via the opt-out letter, another 48 refused to participate when called over the telephone (2 were unreachable), 32 did not arrive to the data collection as scheduled, and 3 did not complete the battery of questionnaires. The authors confirm that all potential participants who declined to participate or otherwise did not participate were not disadvantaged in any way by not participating in the study.

Sleep data were obtained via the School Sleep Habits Survey (SHS) [22]. Aspects of the SHS reported here include UASC sleeping patterns for bed times (BT), rise times (RT), sleep onset latency (SOL), and total sleep time (TST) for schools nights (i.e. nights when there is school on the following day) and weekends (i.e. nights when there is no school on the following day). The SHS is one of the most widely used subjective questionnaire for sleeping patterns in adolescents and has been validated against more objective sleep measures [23], [24]. The SHS has demonstrated suitability across cultures, including adolescents from Korea, Italy, and North America [22], [24]–[30]. The SHS also contains a question concerning nightmare frequency, measured by one question on a rating scale of 1 [never] to 5 [every night] over a two-week period.

The Reactions of Adolescents to Traumatic Stress (RATS) questionnaire was used to screen for probable PTSD [31]. This is a 22-item, self-report screening tool corresponding to DSM-IV criteria for PTSD, developed and validated for UASC. The RATS has high structural, content, and construct validity (Cronbach’s alpha for the total score = .88 and test – retest reliability stability coefficient = .61). The suggested cut-off score is at the 50th percentile (>50.00). Cronbach’s alpha for the RATS total score in this study was = .86. The RATS contains three sleep-related questions concerning nightmares, problems falling asleep, and trouble staying asleep or waking early. RATS is the most valid PTSD screening instrument available for UASC as it was developed for these children and validated against multiple other populations [32].

Demographic data were provided by the local authority social services and UK Border Agency. All questionnaires were translated into Pashto and Dari using double-blind back translations [33]. Interpreters were present at questionnaire administration. This research is compliant with the standards recommended by the Strengthening the Reporting of Observations Studies guidelines [34].

### Data Analysis

Descriptive analyses were performed to check for demographic differences between participant and non-participant groups using χ^2^ and Mann-Whitney (U). Non-parametric Wilcoxon Signed Rank statistical tests were performed to check for differences between BT, RT, SOL, and TST for school nights and weekends and Spearman’s Rho Correlation Coefficient for correlations between sleeping patterns, nightmares, and RATS total score, conforming to the suggested procedures for non-normal data [35]. ANOVA were performed to check for group differences in sleeping patterns between UASC scoring above and below the suggested RATS cut-off score. Where the ANOVA assumption of homogeneity of variance was not met, the robust Welch test (V) was performed [36]. A value of p<.05 was considered statistically significant. All analyses were conducted using SPSS version 18 for Mac.

## Results

### Description of the Sample

Table 1 summarises the demographic statistics. No significant differences were found between participating and non-participating UASC. Reasons for UASC non-participation were unknown, as the children either explicitly refused or did not attend the data collection session. Approximately two-thirds (63.1%) of all UASC lived in a foster home, while the rest lived in semi-independent care (36.9%). UASC reported a mean of 2.15 placements (SD = 1.34), reflecting approximately one change in potential sleeping place. Mean total score on the RATS was 45.7 (SD = 10.9) and approximately one-third (34.3%) of the children scored above the suggested cut-off. A full discussion of PTSD in this sample is reported in a companion paper [37].

**Table 1:** Demographic statistics of the sample.

	Total Group	Participants	Non-participants	
	N = 326	n = 222 (68%)	n = 104 (32%)	
**Socio-Demographic Characteristics**				
** Male**	100%	100%	100%	
** Age Mean (SD)**	16.34 (1.05)	16.34 (1.03)	16.36 (1.08)	U = 11183.0, ns
** Age Range**	13.14–17.97	13.14–17.97	13.16–17.97	
** First Language** a				χ^2^ = .237, ns.
** Pashto**	231 (70.9%)	155 (69.8%)	76 (73.1%)	
** Dari**	94 (28.5%)	66 (29.7%)	28 (26.9%)	
** Farsi**	1 (0.6%)	1 (0.5%)	0 (0.5%)	
** Time in Country**				U = 10543.40, ns
** Mean in days (SD)**	588 (391)	572 (391)	624 (390)	
** Range**	3–1855	3–1776	25–1855	

a When examining first language, assumption ii for χ2 test was not met, as there were only N = 2 Farsi speakers. As no more data would be collected, it was decided to remove the Farsi case and rerun the analysis, χ2 = .237, ns.

### General Sleeping Patterns

Complete information on sleeping patterns (BT, RT, SOL, TST) was available for 189 questionnaires (85.1%) and is summarized in Table 2. Comparing school night and weekend sleep patterns, significant differences were found between bed times, such that UASC went to bed approximately 1.5 hours before midnight on school nights compared to just before midnight on weekends (Z = −11.476, p<.001). Significant differences were also found between waking up times, whereby on school nights UASC reported waking up at approximately 7.00AM compared to 9.45AM on weekends (Z = −11.890, p<.001). Finally, UASC reported sleeping just over one hour less on school nights compared to weekends (Z = −7.885, p<.001). No significant differences were reported for sleep onset latency between school nights and weekends. Nightmares over a two-week period were reported by 64.0% of the young people with the following frequencies: 1–2 times (34.2%), 3–4 times (13.1%), 5–6 times (10.8%), or everyday (5.6%).

**Table 2:** Self reported total sleep time, bed times, and rise times for Afghan UASC on school nights and weekends and significant differences between the two times.

N = 189	Bed time (SD)	Rise Time (SD)	TST (SD)	Sleep Onset Latency (SOL)
School Night	22∶25 (1.19 hours)*	7∶02 (57 minutes)**	7.87 hours (1.5 hours)***	46 minutes (33 minutes)
Weekend	23∶55 (1.39 hours)	9∶43 (1.73 hours)	9.02 hours (2.00 hours)	47 minutes (36 minutes)
Difference (WE – SN)	1.50 hours	2.68 hours	1.15 hours	1 minute

* Z = −11.476 p<.001;

** Z = −11.890 p<.001;

*** Z = −7.885 p<.001; notes: any times over 60 minutes have been calculated into hours. Minutes have been rounded off to the nearest minute.

### Correlation of PTSD with Sleeping Patterns and Nightmares

This section presents findings concerning the correlation of PTSD scores with general sleeping patterns for the full sample. Positive significant correlations were found for probable PTSD symptom scores with bed time (p<.001) and sleep onset latency (p<.001) on school nights, and with bed time (p = .007) and sleep onset latency (p<.001) on weekends. Negative significant correlations were found for probable PTSD symptoms with total sleep time on school nights (p<.001) and on weekends (p = .008). Similarly, a positive significant correlation was identified between nightmare frequency and probable PTSD symptoms (p<.001). Significant correlations were not found between PTSD scores and rise times on school nights (p = .780) or on weekends (p = .643). Full results are presented in Table 3.

**Table 3:** Correlation of RATS total score with sleeping patterns and nightmares.

		School Nights				Weekends				
	Spearman’s rho	Bed time	Risetime	Total Sleep Time	Sleep Onset Latency	Bed time	Risetime	Total Sleep Time	Sleep Onset Latency	Nightmare Frequency
**RATS Total Score**	Correlation Coefficient	.237**	−0.019	−.313**	.354**	.183**	−0.032	−.187**	.319**	.354**

** Correlation is significant at the 0.01 level (2-tailed).* Correlation is significant at the 0.05 level (2-tailed).

### Group Differences in Sleeping Patterns between PTSD and no-PTSD Groups

This section compares sleeping patterns of the PTSD and no PTSD groups. On school nights, UASC with PTSD reported bed times that were half-an-hour later compared to those without PTSD [F (1,218) = 8.810, p = .03], took 20 more minutes more to fall asleep [F (1,197) = 15.09, p<.001], and had a TST of an hour less [F (1,195) = 19.64, p<.001]. On weekends, UASC with PTSD took just over a quarter-of-an-hour more to fall asleep [F (1,200) = 8.83, p = .03], and had a TST of 41 minutes less [V (1,103.348) = 4.562, p = .035]. UASC scoring above the cut-off for PTSD also reported significantly higher frequency of nightmares [V (1,115.159) = 12.75, p = .001]. No significant group differences were found for rise time on school nights and weekends or bed times on the weekends (Table 4).

**Table 4:** ANOVA results of total sleep time, bed times, rise times, sleep onset latency for school nights and weekends along with nightmare frequency.

	Non PTSD	PTSD
**School night**		
** Bed Time (SD)** **	22∶13 (1.18 hours)	22∶49 (1.26 hours)
** Rise Time (SD)**	7∶08 (1.07 hours)	6∶52 (1.00 hour)
** SOL mean (SD)** ***	41 minutes (32 minutes)	60 minutes (38 minutes)
** TST mean (SD)** ***	8.15 hours (1.37 hours)	7.15 hours (1.71 hours)
**Weekends**		
** Bed Time (SD)**	23∶49 (1.39 hours)	24∶05 (1.40 hours)
** Rise Time (SD)**	9∶47 (1.70 hours)	9∶28 (1.72 hours)
** SOL mean (SD)** **	42 minutes (36 minutes)	59 minutes (39 minutes)
** TST mean (SD)** *	9.22 hours (1.79 hours)	8.53 hours (2.27 hours)
**Nightmare frequency over a two-week period** ***		
** Don’t know**	2	6
** Never**	61	11
** 1–2 times**	53	23
** 3–4 times**	16	13
** 5–6 times**	11	13
** Every day/night**	4	9

* = p<.05;

** p<.01;

*** p<.001.

## Discussion

The primary aim of this study is to examine the sleeping patterns of Afghan UASC in the UK, and the relationship between their self-reported sleep and PTSD symptoms. The bed times and rise times of Afghan UASC appear consistent with those of adolescents from other Western or high-income countries (Table 5) [26], [27], [29], [30], [38]. These results suggest, perhaps, some pattern of sleep acculturation among the young people. While taking account of traditional sleep practices, Afghan UASC sleep patterns appear to adapt to their contextual environment, which includes social and cultural norms of the country of asylum [39], [40]. Bed times might be associated with the socialising patterns of UASC. With increased time spent in the country of asylum these children are likely to associate non-UASC peers, and that their bed times would thus reflect certain typical patterns, such as staying out late on weekends. Rise time acculturation is most likely a reflection of waking up for school.

**Table 5:** Comparison of means for TST, BT, RT, Oversleep, and Bed time Delay from a selection of other studies using the SHS.

			School Nights			Weekends		
	N	Age^a^	TST	Bed time	Rise Time	TST	Bed time	Rise Time
			(SD)	(SD)	(SD)	(SD)	(SD)	(SD)
**Afghan UASC UK (no PTSD)**		16.3	489	22∶13	7∶08	553	23∶49	9∶47
			(82)	(71)	(64)	(107)	(83)	(102)
**Afghan UASC UK (PTSD)**			429	22∶49	6∶52	512	24∶05	9∶28
			(102)	(76)	(60)	(136)	(84)	(103)
**USA ** [**29**]	336	13–14	462	22∶05	5∶59	567	23∶54	9∶22
			(67)	(49)	(24)	(100)	(94)	(85)
	858	15	449	22∶20	6∶00	564	0∶06	9∶40
			(66)	(55)	(25)	(104)	(83)	(104)
	919	16	435	22∶37	6∶05	549	0∶30	9∶46
			(68)	(58)	(29)	(108)	(82)	(107)
	988	17–19	424	22∶51	6∶10	518	0∶49	9∶32
			(66)	(58)	(31)	(114)	(80)	(107)
**Italy ** [**26**] b	E-Types = 742	14.1–16^a^	460	23∶05	7∶10	545	1∶15	10∶55
	M-Types = 995	14.1–16^b^	490	23∶30	6∶40	535	23∶40	8∶55
		16.1–18.6^a^	440	23∶30	7∶10	530	2∶25	11∶25
		16.1–18.6^b^	480	23∶30	6∶25	500	00∶25	09∶10
**Korea ** [**30**]	1457	Grades 5–6	492	22∶42	7∶18	534	23∶06	7∶54
			(72)	(96)	(30)	(150)	(138)	(96)
		Grades 7–8	456	23∶12	7∶00	486	23∶12	8∶24
			(60)	(72)	(30)	(162)	(186)	(108)
		Grades 9–10	396	0∶06	6∶54	498	23∶00	9∶30
			(84)	(72)	(36)	(168)	(288)	(108)
		Grades 11–12	324	0∶54	6∶18	492	0∶24	9∶12
			(102)	(96)	(24)	(126)	(120)	(102)
**USA ** [**27**]	89	11.6	552	21∶32	6∶39	552	23∶16	8∶49
			(78)	(70)	(35)	(120)	(79)	(118)
**USA ** [**38**]	225	16.4	427	23∶39	6∶54	572	0∶56	10∶22
			(46)	(51)	(29)	(106)	(63)	(78)

TST and standard deviations reported in minutes.

a Mean Age/age range/school grade.

b Standard deviations not reported.

What warrants greater investigation for UASC is the time to fall asleep after going to bed, and how this may be used as a potentially less culturally contested method of assessment for distress. The mean sleep onset latency for all UASC interviewed in this study was over 45 minutes (Table 2), the sole sleeping pattern variable not significantly different between school nights and weekends. By comparison, the sleep onset latency mean found in the US National Sleep Survey was approximately 25 minutes among adolescents aged 11–17, nearly half the sleep onset latency time for all Afghan UASC in this study [41]. It appears that UASC sleep onset latency is nearly 20 minutes greater than what may be considered a normative length of time to initiate sleep [42]. Thus, it may be that the 25 minute sleep onset latency mark suggested above is inappropriate for UASC when considering their pre and post migration experiences and that the mean sleep onset latency for the whole group indicated here, 45 minutes, is a more contextually valid number. Comparison with other UASC groups or Afghan children in Afghanistan is not possible however due to the lack of any evidence to date.

Expectedly, there is a strong association between increased sleep problems and scoring above the cut-off for PTSD on the RATS among the children in this study in terms of nightmare frequency, but also in terms of increase sleep onset latency time. UASC who scored above the cut-off for PTSD reported greater sleep onset latency by at least 20 minutes compared with the non-PTSD group. As such, the 60-minute sleep onset latency mean found for UASC who scored above the cut-off for PTSD should be considered problematic.

The assessment of sleep on its own however may have direct implications for the clinical assessment of distress in UASC. Arguably, the assessment of sleep problems can allow for a less culturally biased approach to screening for PTSD within this group, as much of the arguments centre on the reliability and validity of diagnostic and screening instruments for mental health across cultures [43]–[45]. Questions about sleep may be more acceptable and understandable concepts for assessment across cultures and serve as strong predictive markers for distress. All major mood disorders in the DSM-IV have sleep problems as one of their cardinal features, for example Depression and Anxiety disorders. In children there is considerable literature concerning the confusion of sleeplessness and behavior problems [46].

The approach to UASC distress through the assessment of sleep presents a potentially more culturally appropriate method for the assessment of UASC distress and, importantly, provides a novel direction for interventions. The assessment of UASC sleep problems with direct questions concerning sleeping patterns or perhaps sleep diaries may provide non-intrusive, culturally neutral forms of understanding if the child is sleeping enough, waking up at night for specific reasons, or having disturbing images in their sleep (i.e. nightmares). This is not discounting the important argument on different sleep ecologies across cultures as made by Worthman and Melby (2002). Similarly, the assessment of UASC sleep patterns in the country of asylum would duly consider the ‘Western, post-industrial’ context of their current lives.

Intervention research concerning UASC and other refugee children is generally limited and there is no known evidence of interventions for UASC sleep disturbances [47]–[49]. The impact of trauma on sleep and the centrality of sleep problems as a feature of PTSD, however, strongly suggest that this should be an area of increased research. Indeed, targeting disturbed sleep for traumatised UASC may yield results such as decreased levels of PTSD alongside improved sleep [50], [51].

### Limitations

There are several limitations of this study that need to be addressed. The cross-sectional study design and non-participation of nearly 30% of the potential sample may ultimately limit the understanding of how the sleeping patterns of these children may develop or change the longer they remain in the UK. Cross-sectional studies are often used to provide information concerning problems within a community. The large sample size of this study provides a strong indication of sleeping patterns and provides increased external validity. Individuals who elected not to participate in this study may have had higher levels of distress, indicating some self-selection bias. Comparisons of demographic, care, and asylum variables between participants and non-participants found no significant group differences.

Another limitation is the lack of comparison group either by immigration status, living arrangements, or other countries of origin. This is an exploratory study examining sleep patterns within a particularly population, and comparing them to historical controls using the same measures. While this study lays the initial groundwork, further research comparing UASC to contemporary controls is necessary. This study sample comprises a relatively homogenous group of UASC, thus removing the variable of mixed populations that has confounded previous studies. Doing so has improved the clarity of analyses of sleep and PTSD in a way that has not been previously possible and is a particular strength of this work.

Self-report methods were applied in the collection of information concerning mental health and sleep, thus limiting results to the subjective estimations of the young people. The SHS has been validated against actigraphy and sleep diaries, both more objective assessments of sleep patterns.

A final limitation concerns the cultural mediation and translation of mental health concepts. Concepts and ideas may not transfer clearly across cultures and this may result in the decreased validity of screening instruments [44]. Extensive efforts were made to minimise any potential translation and interpretation bias. As stated above, all questionnaires were translated through double-blind back techniques and interpreters were present at questionnaire administration.

### Conclusion

This study presents the first systematic approach to understanding the sleep of UASC. The study sample size allows for a strong, contextually clear picture of Afghan UASC sleeping patterns. Importantly, the findings suggest that sleep assessments could be used as culturally appropriate methods for detecting UASC distress and that consideration should be given to interventions targeting sleeping problems as possible approaches to treating this distress.
